# Anticoagulation for patients with mechanical heart valves at the end of life: understanding clinician attitudes and improving decision making

**DOI:** 10.1186/s12904-021-00809-z

**Published:** 2021-07-16

**Authors:** Jonathan Raby, Victoria Bradley, Nikant Sabharwal

**Affiliations:** 1 University Hospitals Birmingham NHS Foundation Trust, Mindelsohn Way, Birmingham, B15 2TH UK; 2 Oxford University Hospitals NHS Foundation Trust, Headley Way, Headington, Oxford, OX3 9DU UK

**Keywords:** Survey, Mechanical heart valves, Anticoagulation, End of life, Decision support

## Abstract

**Background:**

Decisions regarding continuation or cessation of anticoagulation for patients with mechanical heart valves nearing the end of life represent a difficult balance of risks. The risk of suffering and disability that may result from thromboembolism must be weighed against the burden of continued anticoagulation therapy and the excess bleeding risk this confers. Data allowing quantification of the relative risks are scarce, and this translates to a lack of published guidance on the topic. Here we describe how this lack of guidance is impacting upon healthcare professionals and their patients through misconception of risk and under-confidence in decision-making. We also present local guidance we have developed that aims to improve objective risk assessment and promote individualised, patient-centred decision-making.

**Methods:**

Our survey was developed by specialists in palliative care and cardiology. The survey explored respondents' conception of the risks of stopping anticoagulation for patients with mechanical heart valves at the end of life, as well as their ability to identify patient factors that modify this risk. Respondent decision-making, confidence, and readiness to accept further guidance were also explored. Healthcare professionals at two university teaching hospitals were invited to participate in the survey. The study population included hospital specialists, generalists, and trainees.

**Results:**

Fifty-two healthcare professionals completed the survey, including 16 palliative care specialists. 47 (90%) of respondents felt poorly informed of the risks of stopping or continuing anticoagulation. 6 (12%) correctly identified risk of thromboembolism in patients with mechanical heart valves who are not anticoagulated. The remainder overestimated risk by a factor of two (18, 35%) or five (27, 52%). 49 (94%) would find further guidance on this issue helpful.

**Conclusions:**

The healthcare professionals we surveyed felt poorly informed and ill-equipped to make decisions regarding anticoagulation for patients with mechanical heart valves at the end of life. They were objectively poor at estimating the risks involved. In the absence of robust data to support protocolisation of practice, we believe these decisions must be taken in conversation with the patient, taking account of individual circumstances and priorities. We have developed guidance for local use to support such individualised decision-making.

## Background

Valvular heart disease is a significant cause of morbidity in the developed and developing world, and its prevalence increases linearly with age [3]. Mechanical heart valve replacement remains an important therapeutic option for a carefully selected subset of patients [9]. ‘Lifelong’ anticoagulation is generally advocated for such patients and despite the advent of direct-acting oral anticoagulants, Warfarin and heparins remain the therapeutic agents of choice [4]. As the global population ages, the number of patients living and dying with mechanical heart valves will increase.

‘End of life care’ describes the process of identifying and addressing physical, psychological, emotional, and social needs of patients in the last 12 months of life [6]. As patients reach the last weeks or days of life, an important component of this care is review and agreed upon cessation of medications that are no longer providing benefit or may cause harm [7]. Applying this framework to the anticoagulation of patients with mechanical heart valves represents a particular challenge. Warfarin monitoring and administration of heparins can be burdensome, while bleeding risk is increased in a population with a relatively high prevalence of hepatic and renal dysfunction. These factors must be weighed against the distress and disability that might be conferred by thromboembolic events.

Data defining the risks of stopping anticoagulation are scarce, and many studies that demonstrate the efficacy of anticoagulation in preventing thromboembolism date back to the 1960s [2]. Modern valve prostheses are less thrombogenic and are likely to pose less of a risk in the absence of anticoagulation, although absence of clinical equipoise has prevented empiric confirmation of this. Furthermore, the validity of applying data quantifying long-term risk to patients in the last days or weeks of life is questionable, and clinical trials specific to the palliative care population are rare [8]. Observational data on the risk of thromboembolic events on cessation of anticoagulation under various circumstances (Table 1) suggest that short term thrombosis risk may be rather lower than traditionally estimated [1]. For example, the incidence of thrombosis associated with a St Jude bi-leaflet mechanical aortic valve prosthesis in the absence of anticoagulation has been estimated at 0.03% per day. For patients whose life expectancy is in the region of days to short weeks, this may support a decision to stop anticoagulation therapy.

**Table 1:** Risk of thromboembolic events in the absence of therapeutic anticoagulation

	**Risk of Thromboembolism Without Anticoagulation**	
	**Annualised Risk (%)**	**Daily Risk (%)**
**Lone AF**	1	0.003
**Average risk AF**	5	0.014
**High risk AF**	12	0.033
**AVR (St Jude)**	11	0.03
**AVR (B-Shiley)**	23	0.06
**MVR (St Jude)**	22	0.06
**Multiple VR**	91	0.25
**Previous VTE**	4	0.01
**VTE in last month**	-	1.6

Adapted from [1]

The absence of good quality data translates to a lack of consensus and guidance on how to make decisions regarding anticoagulation at the end of life for patients with mechanical heart valves. Here we present the results of a survey of healthcare professionals that demonstrates uncertainty and misconception of risk in the approach to such patients. We also present a local guideline that we have developed to support decision making.

## Methods

**Fig. 1:**
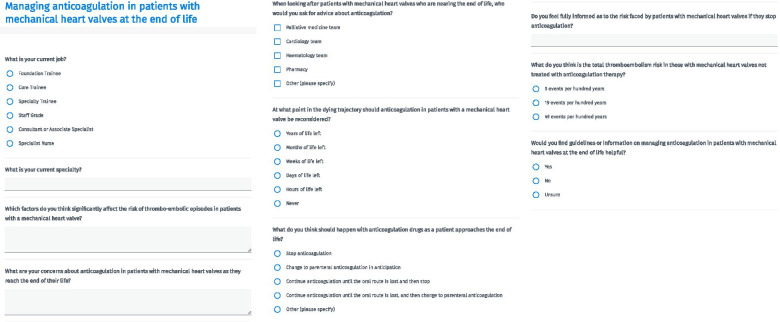
Survey developed and circulated to explore healthcare professional attitudes and understanding of the issues surrounding anticoagulation for patients with mechanical heart valves at the end of life

A survey was designed to assess healthcare professionals' confidence and understanding when making decisions regarding anticoagulation at the end of life (see Fig. 1 for the survey in full). Questions were drafted by consultants in palliative care and cardiology, and the survey was refined following feedback from a focus group that was representative of the population to be surveyed. This survey was not formally validated. Respondents were asked to estimate the risk of thromboembolism in patients with mechanical heart valves in the absence of anticoagulation, and were asked to list factors that affect this risk for individual patients. Responses were reviewed for correct identification of risk factors as described in the literature (including type of valve, position of valve, and concomitant atrial fibrillation). Respondents were asked whether they felt confident in making decisions regarding anticoagulation for patients with mechanical heart valves at the end of life, whether they would find guidance on this topic useful, and which hospital specialists they would ask for advice in the absence of such guidance.

Healthcare professionals were invited to participate if their current area of practice brought them into contact with patients with mechanical heart valves approaching the end of life. Consultants, specialty trainees, and specialist nurses in palliative care were included, as were Consultants and junior doctors working in general medical inpatient settings across two university teaching hospitals. This survey population was designed to assess specialist and non-specialist attitudes. The survey was circulated to target groups via SurveyMonkey®, and participation was voluntary. Results were analysed using Microsoft Excel®.

## Results

Of 310 healthcare professionals to whom the survey was circulated, 52 (17%) responded. This is in line with typical online survey response rates reported in the literature [5, 10]. Of these, 36 (69%) were foundation doctors, 10 (19%) were consultant physicians, 4 (8%) were specialist nurses and 2 (4%) were specialty trainee doctors. 16 (31%) were palliative care medicine specialists, while the remainder worked in other hospital medical specialties. 47 (90%) of respondents felt they were not well informed of the risks associated with stopping anticoagulation for patients with mechanical heart valves at the end of life, and this included 100% of palliative care specialists. 49 (94%) stated that they would find guidance on this issue helpful. In the absence of such guidance, there was no consensus on which hospital team should provide advice– 19 (37%) would ask haematology, 15 (29%) cardiology, and 15 (29%) palliative care, while 3 (6%) would ask a combination of these teams.

6 (12%) respondents correctly identified risk of thromboembolism in patients with mechanical heart valves who are not anticoagulated, with the remainder overestimating risk by a factor of two (18, 35%) or five (27, 52%). 14% of foundation doctors correctly estimated the risk, compared with 10% of consultants, and 6% of palliative care specialists (comprising consultants, registrars, and specialist nurses).

32 (62%) were able to identify at least one factor that affects risk of thromboembolism in patients with mechanical heart valves (mean number of risk factors identified 0.81, SD 0.76).

When considering a decision to stop anticoagulation, 30 (58%) would favour continuation of anticoagulation until the oral route of administration was lost, with a further 4 (8%) stating that parenteral anticoagulation should be continued until death once the oral route is lost. 21 (40%) respondents stated that they would reconsider an anticoagulation prescription once a patient entered the last days of life, while 19 (37%) would review in the last weeks of life, 11 (21%) in the last months of life, and 1 (2%) in the last hours of life.

## Discussion

Deciding to stop anticoagulation for patients with mechanical heart valves at the end of life represents a difficult balance of risks, and it is a decision that must currently be informed by incomplete and indirectly relevant data. In this study, we have described how the lack of local or national guidance on this subject is impacting upon the attitudes and decision-making of healthcare professionals. We found that most (including palliative care specialists) feel ill-informed to make this decision, and were objectively poor at identifying patient factors that should be considered. That these findings were consistent across foundation doctors and consultants in palliative care demonstrates that neither recency of medical education nor personal clinical experience make a significant difference to preparedness.

This suggests the need for better guidance, and we found the overwhelming majority of healthcare professionals would be receptive to this. While acknowledging the paucity of quality data available to allow estimation of thromboembolic risk in the absence of anticoagulant therapy, we believe that such data as do exist support at least discussing cessation of anticoagulation with patients entering the last days or weeks of life, even for subgroups traditionally considered at 'high risk' of thromboembolic events (such as patients with mechanical mitral valve prostheses).

The results of our study suggest that clinicians tend to over-estimate thromboembolic risk for mechanical heart valves and favour continuation of anticoagulation, typically for as long as the oral route of administration is available. Given the potential morbidity associated with a thromboembolic event, it is of course desirable that healthcare professionals approach decisions regarding cessation of anticoagulation with consideration and appropriate reticence. However, harm may also result from inappropriate prolongation of anticoagulation therapy, and we believe that in the absence of convincing data to support anticoagulation continuation or cessation this decision should be taken on a case-by-case basis following careful discussion with the individual patient.

**Fig. 2:**
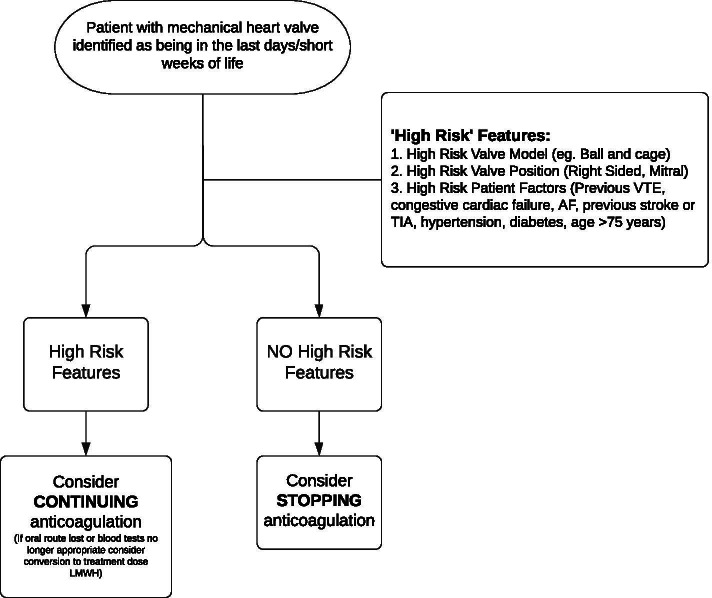
Summary 'decision aid' from local guideline on managing anticoagulation for patients with mechanical heart valves approaching the end of life

We believe, therefore, that a useful guideline will empower healthcare professionals to initiate these conversations with patients they have identified to be approaching the end of life. We have developed a guideline locally for those approaching this complex issue that encourages individualised decision making. This guideline includes a summary of best available data regarding thromboembolic risk on cessation of anticoagulation for a variety of patient groups as well as a decision-making tool to provide a more structured way of assessing individual risk for patients with mechanical heart valves (Fig. 2).

## Conclusions

An important component of 'end of life care' is the review and agreed upon cessation of medications that are no longer providing benefit, or that may cause harm. Making this decision with respect to anticoagulation for patients with mechanical heart valve prostheses is particularly daunting due to the perceived high risk of thromboembolism in this cohort, and the excess suffering that may result from such events. This decision is further complicated by a lack of guidance on how it should be approached. Data to inform this decision are scarce, but where available suggest that when adjusted to a timescale relevant to patients in the last days or weeks of life, thromboembolic risk may in fact not be high enough to justify continuation of anticoagulation under all circumstances.

Our study demonstrates that healthcare professionals lack confidence in making such decisions, are objectively poor at assessing the relevant risks for a given patient (with a consistent tendency to over-estimate thromboembolic risk), and may therefore be exposing patients to harm.

We suggest that although empiric data to inform decisions relating to anticoagulation cessation in patients with mechanical heart valve prostheses at the end of life are limited, patient care could still be improved by the development of guidance drawing on expert opinion and available observational data. We have developed a guideline for local use that aims to empower healthcare professionals to initiate conversations with patients regarding their anticoagulation as they approach the end of life, with the objective of reaching an informed decision that is tailored to the individual patient's circumstances and wishes.

## Data Availability

The datasets used and/or analysed during the current study are available from the corresponding author on reasonable request.
